# Improving Glycerol Electrooxidation Performance on Nanocubic PtCo Catalysts

**DOI:** 10.1021/acsami.4c10219

**Published:** 2024-10-14

**Authors:** Irina Terekhina, Mats Johnsson

**Affiliations:** Department of Materials and Environmental Chemistry, Arrhenius Laboratory, Stockholm University, Stockholm SE-106 91, Sweden

**Keywords:** PtCo, nanocubes, glycerol electrooxidation, selectivity, lactate

## Abstract

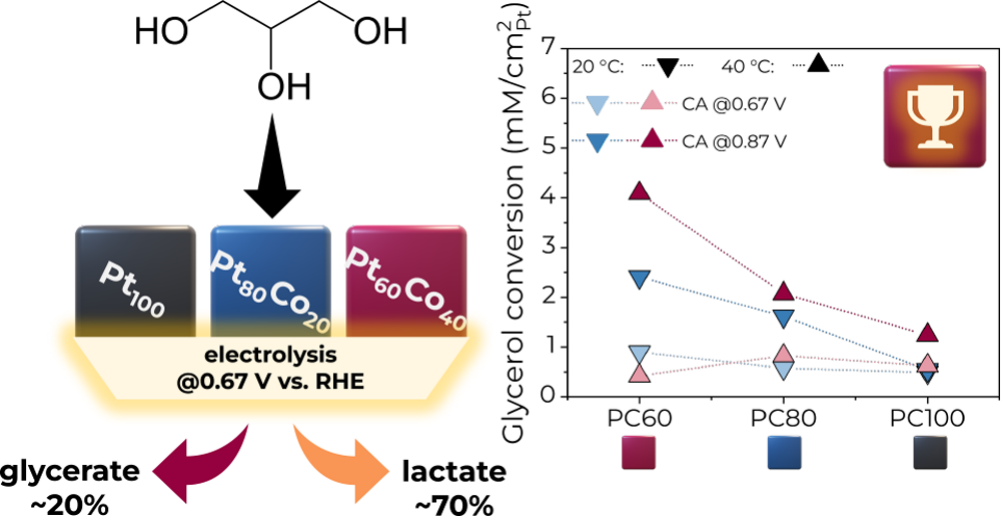

As glycerol (GLY) has emerged as a highly functional and cheap platform molecule and as an abundant biodiesel production byproduct, possible conversion methods have been investigated. One of the promising approaches is the glycerol electrooxidation (GEOR) on noble metal-based catalysts. Although noble metals, especially Pt, are generally very stable at different pH and highly selective toward three-carbon (C3) products, their electrocatalytic performance can be further improved by morphology tuning and alloying with non-noble metals like Co. In the present study, cubic Pt_*x*_Co_100–*x*_ (*x* = 100, 80, and 60) nanoparticles were investigated in an alkaline medium at 20 and 40 °C. The effect of the composition and reaction conditions on the selectivity of the GEOR toward C3 products like lactate and glycerate was studied, and the reaction mechanism was discussed. The highest mass activity was found for Pt_80_Co_20_, although when the specific activity, glycerol conversion, and GEOR selectivity were compared, Pt_60_Co_40_ was the superior catalyst overall. In general, all catalysts, even those that are Co-rich, exhibited a high C3 product selectivity up to 95% at 0.67 V vs RHE. The low applied potential of 0.67 V vs RHE at 40 °C facilitated lactate formation with selectivity up to 72%. At the same time, the glycerate formation with a selectivity of up to 40%, as well as C–C bond cleavage, was more favored at 0.87 V vs RHE.

## 1 Introduction

In recent decades, there has been a noticeable shift in how we want to extract energy, with an emphasis on cleaner, renewable, and sustainable energies and fuels. As a result, biodiesel, a biofuel derived from animal fats, vegetable oils, and their residual materials through catalyzed transesterification, has emerged as such a solution. However, the exponential growth of the biodiesel industry has led to an unprecedented surplus of glycerol (GLY), a byproduct constituting approximately 11 wt % of the total biodiesel production, resulting in nearly 110 kg of crude glycerol per 1 ton of biodiesel generated.^1^ This increasing oversupply has caused a significant decline in the market value of glycerol despite its numerous applications in different areas, thus making it an attractive candidate for further valorization.^2,3^

Within this context, among other approaches,^3−8^ the glycerol electrooxidation reaction (GEOR) has appeared as a promising route for the valorization of glycerol, offering a dual benefit of generating value-added compounds at the anode while concurrently generating hydrogen gas at the cathode, all at a reduced operating cell potential compared to conventional water splitting processes used for hydrogen gas production.^9^ The GEOR products include compounds such as aldehydes, ketones, and carboxylic acids. Among them, three-carbon (C3) products, glyceraldehyde (GALD), dihydroxyacetone (DHA), glyceric acid/glycerate (GLE), hydroxypyruvic acid/hydroxypyruvate (HYDP), lactic acid/lactate (LACT), and tartronic acid/tartronate (TART), are of particular interest due to their versatile applicability and potential economic significance.^10−14^

Extensive research has been devoted to solving the complex mechanism of the GEOR on catalysts, ranging from noble metals to non-noble metals and their various alloys. Noble metal-based catalysts, notably platinum, palladium, and gold, have demonstrated remarkable activity, stability, and selectivity toward C3 products. Moreover, incorporating other metals into a noble metal-based catalyst has shown impressive results in improving the electrocatalytic performance through synergistic effects and changes in electron transfer for the GEOR.^6,11,15−17^ As increasing the number of active sites is one of the ways to enhance the GEOR performance, the design of various nanomaterials as potential catalysts has become a hot topic. In addition to that, the morphology and exposed facets of these catalysts have shown a great effect on the performance and selectivity of the GEOR, and catalysts with dominant {100} faces have been proven to exhibit impressive catalytic characteristics.^18−29^

Platinum and platinum-based catalysts have been studied the most, as platinum has great catalytic performance toward the GEOR and stability in both acidic and alkaline media.^6,17^ Nevertheless, the research has focused mostly on an alkaline medium—it has been shown that alkaline conditions are preferable for the GEOR as the reaction kinetics are more favorable, and a choice of a catalyst is not limited to noble metals, as non-noble metals are unstable in acidic media. Additionally, it has been proposed that the first deprotonation step in the GEOR mechanism is base-catalyzed.^30,31^

Therefore, the design and application of Pt-based bimetallic or trimetallic nanocatalysts for the GEOR in alkaline media have been ongoing for several years, with the alloying elements being presented by Fe, Co, Ni, Ag, and Cu and other noble metals, such as Pd, Au, Rh, and Ru. Various morphologies of Pt-based nanocatalysts, including irregular nanoparticles (NPs),^32−38^ and morphology-tuned nanostructures like porous,^39^ dendritic,^29,40−42^ nanowires,^43^ cubic,^28,29,44,45^ octahedral,^28,46^ and hexagonal nanoparticles^47^ have been investigated. However, the list of publications focused on the performance of noble-metal-based shape-controlled nanocatalysts is scarce, not to mention their alloying with other transition metals. Moreover, only a handful of studies^28,29,32,33,36,38,39,44^ explored the selectivity of the GEOR in addition to widely reported electrocatalytic characteristics such as current density, mass activity, and specific activity.

To our knowledge, no prior work has been reported using cubic Pt–Co nanocatalysts with dominant {100} faces, featuring a thorough quantitative evaluation of the GEOR products at different reaction conditions. Herein, we report a study on the GEOR selectivity and electrocatalytic performance of cubic Pt_*x*_Co_100–*x*_ (*x* = 100, 80, and 60) NPs in an alkaline medium. Besides the composition, the effects of such parameters as applied potential and reaction temperature were studied. The morphology and composition of the catalysts were characterized by transmission electron microscopy (TEM), energy-dispersive X-ray spectroscopy (EDS), and powder X-ray diffraction (PXRD). The electrocatalytic activity was evaluated with cyclic voltammetry (CV), and the GEOR was performed in a potentiostatic mode at 0.67 and 0.87 V vs RHE with further GEOR products analysis using high-performance liquid chromatography (HPLC). The present study demonstrates that alloying with Co does not hinder the electrocatalytic performance. On the contrary, the Co-containing catalysts showed an improved activity compared to pure Pt while keeping the C3 product selectivity almost unchanged. Furthermore, the distribution of the GEOR products remained almost unchanged even on Co-rich catalysts, and LACT was the dominant product with a selectivity of up to 70–72%.

## 2 Experimental Section

### 2.1 Chemicals and Materials

Platinum(II) acetylacetonate (Pt(acac)_2_, Acros Organics, 98%), cobalt(II) acetate tetrahydrate (Co(OAc)_2_·4H_2_O, Alfa Aesar, >98%), tungsten hexacarbonyl (W(CO)_6_, Merck, ≥98%), hydrochloric acid (HCl, VWR chemicals, 37%), potassium hydroxide (KOH, Honeywell, puriss. p.a., ≥86%), glycerol (bidistilled, VWR chemicals, ≥99.5%), oleylamine (OAm, Sigma-Aldrich, technical grade, 70%), oleic acid (OA, Tokyo Chemical Industry, >99.0%), hexane (Honeywell, >99%), ethanol (absolute, VWR chemicals, ≥99.7%), isopropanol (Carlo Erba Reagents, ≥99.5%), and Nafion perfluorinated resin solution (5 wt % in lower aliphatic alcohols and water, contains 15–20% water) were used without further purification. Carbon fiber paper (CFP, H23, thickness 210 μm) was purchased from Freudenberg and subjected to hydrophilic treatment and catalyst modification (see below).

Ultrapure water with a resistivity of 18.2 MΩ·cm from Millipore (Simplicity Water Purification System) was used throughout all of the electrochemical experiments and aqueous solution preparations.

### 2.2 Synthesis of Cubic Pt_*x*_Co_100–*x*_ (*x* = 100, 80, and 60) Nanoparticles

The cubic Pt NPs were prepared using a reported procedure^48^ optimized in another study.^29^ To synthesize Pt_80_Co_20_ and Pt_60_Co_40_ NPs, the procedure was further altered and optimized. Similarly, a mixture of 40 mg of Pt(acac)_2_, varying amounts of Co(OAc)_2_·4H_2_O (17.9 and 45.7 mg for Pt_80_Co_20_ and Pt_60_Co_40_, respectively), 32 mL of OAm, and 8 mL of OA in a 50 mL round-bottomed flask equipped with a rubber septum was flushed with argon gas for 15 min to remove excess oxygen while being heated to 130 °C under magnetic stirring of 1000 rpm. Then, 200 mg of W(CO)_6_ was added, followed by an increase in the reaction mixture temperature to 220 °C. After that, the mixture was left to react for 1 h under the same stirring rate and argon stream. Finally, the resulting solution was washed by centrifugation four times with hexane at 12,000 rpm for 15 min, followed by the redispersion in hexane for storage.

### 2.3 Structural Characterization

Transmission electron microscopy (TEM) imaging and energy-dispersive X-ray spectroscopy (EDS) analysis were carried out on a JEOL JEM-2100F microscope operating at an acceleration voltage of 200 kV. TEM samples were prepared by depositing ∼20 μL of nanoparticle hexane dispersion on a 200-mesh carbon-coated copper grid and allowing the solvent to evaporate. The powder X-ray diffraction (PXRD) was performed on a Bruker D8 Discover diffractometer using a Cu Kα X-ray source (λ_CuKα1_ = 1.540598 Å and λ_CuKα2_ = 1.544426 Å), equipped with an LYNXEYE XE-T detector. The diffractograms were collected within the 2θ range of 10–90° with a step size of 0.01° and scan rate of 0.375°/min.

### 2.4 Electrochemical Measurements

Details of carbon fiber paper (CFP) and Nafion membrane treatments, catalyst ink preparation, and working electrode fabrication were previously described by Terekhina et al.^28^

All electrochemical measurements were performed using an SP-50 potentiostat (Biologic, France) at 20 or 40 °C. The electrochemical setup was a typical three-electrode cell with the catalyst-modified CFP electrodes as working electrodes, saturated calomel electrode (SCE) as the reference electrode, and platinum mesh as the counter electrode. Electrocatalytic performance was investigated in a 2 M KOH solution as a catholyte and a 2 M KOH + 1 M GLY solution as an anolyte. All the solutions were freshly prepared before use and purged with argon gas for 30 min before the measurements, which was maintained throughout the experiments.

To stabilize the catalyst surface and calculate the absolute electrochemical surface area (aECSA), 10 cycles were registered using cyclic voltammetry (CV) in the potential range of −1.0 to 0.2 V vs SCE in 25 mL of 2 M KOH solution in a single-compartment electrochemical cell at 20 °C.

The hydrogen underpotential deposition region (*H*_UPD_) was used for the aECSA calculation with eq 1:
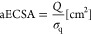
1where *Q* is the total charge corresponding to the hydrogen adsorption/desorption, *C*, and σ_q_ is the charge density for the adsorption of a *H*_UPD_ monolayer, 210 μC/cm^2^.^49^

To study electrocatalytic performance in 2 M KOH + 1 M GLY electrolyte and subsequently perform chronoamperometric measurements (CA), the working and reference electrodes were inserted into the anodic chamber and the counter electrode into the cathodic chamber of the divided electrochemical cell with a total cell volume of 30 mL. The chambers were separated by a Nafion (N-117) membrane. Chronoamperometric measurements were carried out in a potentiostatic mode at 0.67 and 0.87 V vs the reversible hydrogen electrode (RHE) for 2 h with a constant stirring of 500 rpm. The electrocatalytic activity after the CA was assessed in 25 mL of a catholyte or anolyte in a single-compartment cell registering 5 cycles.

In order to further analyze the GEOR products using high-performance liquid chromatography (HPLC), ∼150 μL was sampled from the anolyte and catholyte before and after the electrolysis. Next, 100 μL aliquots of the anolyte and catholyte were neutralized by 100 μL 1 M H_2_SO_4_ and diluted by 800 μL of ultrapure water. The concentration of GLY that migrated to the cathodic compartment was less than 1% of the initial concentration for GLY, and the concentrations of the GEOR products were at the trace level.

All the electrochemical data are presented with an IR correction (at 85% compensation) due to some uncompensated resistance that was in the range of 2.4–4.1 Ω for 2 M KOH and 1.7–3.7 Ω for 2 M KOH + 1 M glycerol electrolytes. The CV curves were recorded at a scan rate of 50 mV/s, and the current was converted to the mass activity (*J*_mass_) and specific activity (*J*_ECSA_) according to eqs 2 and 3:
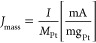
2
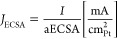
3where *I* is current, mA; *M*_Pt_ is absolute Pt loading: *M*_Pt_ = 1 mg_Pt_, *M*_Pt80Co20_ = 0.927 mg_Pt_, and *M*_Pt60Co40_ = 0.814 mg_Pt_. Reported potentials were converted versus the RHE using eq 4:
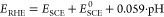
4where *E*^0^_SCE_ = 0.248 V vs the standard hydrogen electrode (SHE) at 20 °C.

Details regarding the total Faradaic efficiency (FE) and carbon balance (CB) calculations are presented in Tables S1–S2 and eqs S1–S4.

### 2.5 HPLC Measurements

The HPLC analysis of GEOP products was performed with an Agilent 1260 Infinity II isocratic pump, multisampler, and multicolumn thermostat equipped with a 1290 Infinity II refractive index detector. The analytical columns connected in series included a Bio-Rad guard column with a standard cartridge holder with a Micro-Guard cation H^+^ cartridge (4.6 × 30 mm), a Bio-Rad Aminex HPX-87H column (7.8 × 300 mm), and a Shodex Sugar SH1011 column (8 × 300 mm). The columns were kept at 30 °C, and the eluents were 1 and 8 mM solutions of H_2_SO_4_. The injection volume was 20 μL, and the sample analysis time was 85 min at a flow rate of 0.25 mL/min.

Details regarding the standards used for the calibration and the calibration curves, together with the HPLC chromatograms of standard solutions, can be found in our previous study.^29^ Briefly, the possible GEOR products for which the analysis was calibrated were the standards of GLY, dihydroxyacetone (DHA), glyceraldehyde (GALD), glycerate (GLE), lactate (LACT), hydroxypyruvate (HYDP), tartronate (TART), mesoxalate (MESOXA), glycolate (GLO), oxalate (OXA), glyoxylate (GLYOXY), acetate (ACET), and formate (FORM), and the retention times (RT) were 66.9, 70.5, 57.6, 56.2, 64.4, 46.8, 45.4, 38.7, 63.4, 38.1, 52.5, 78.8, and 73.5 min, respectively. Note that two mobile phases (1 and 8 mM solutions of H_2_SO_4_) were used for better peak separation of glycerol, glycerate, lactate, and glycolate. Thus, their RTs noted above were derived from HPLC analysis in 1 mM H_2_SO_4_. In addition to the electrolysis samples, 2 M KOH samples were tested to identify peaks not associated with any of the GEOR products.

## 3 Results and Discussion

### 3.1 Structural Characterization of Pt_*x*_Co_100–*x*_ (*x* = 100, 80, and 60) Nanoparticles

**Scheme 1:**
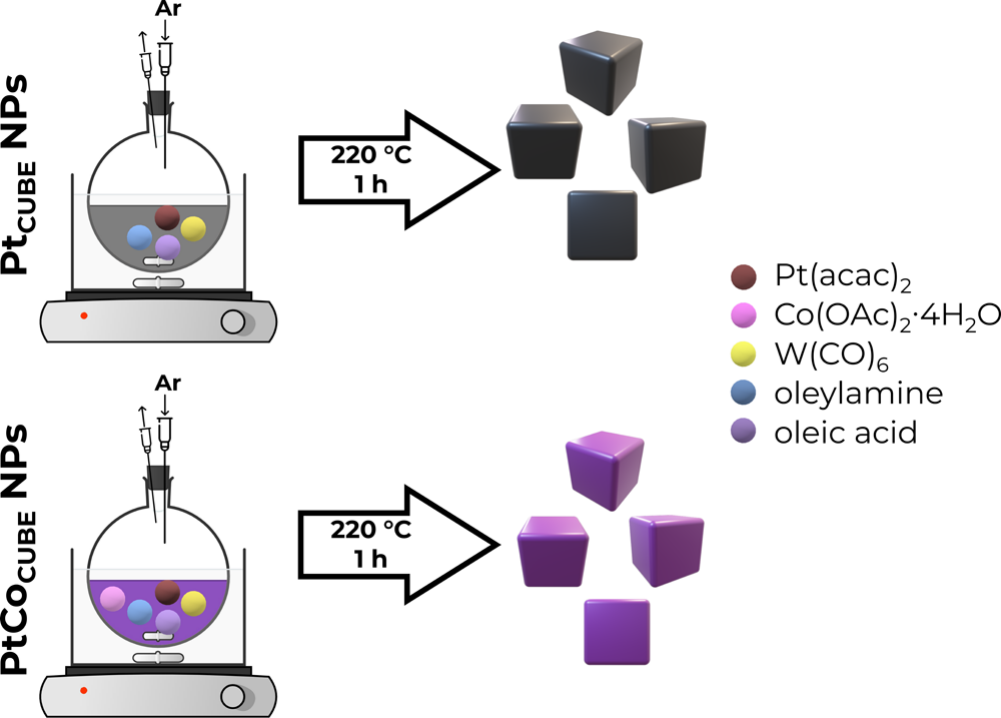
Schematic Representation of the Pt_*x*_Co_100–*x*_ NPs Synthesis

**Figure 1:**
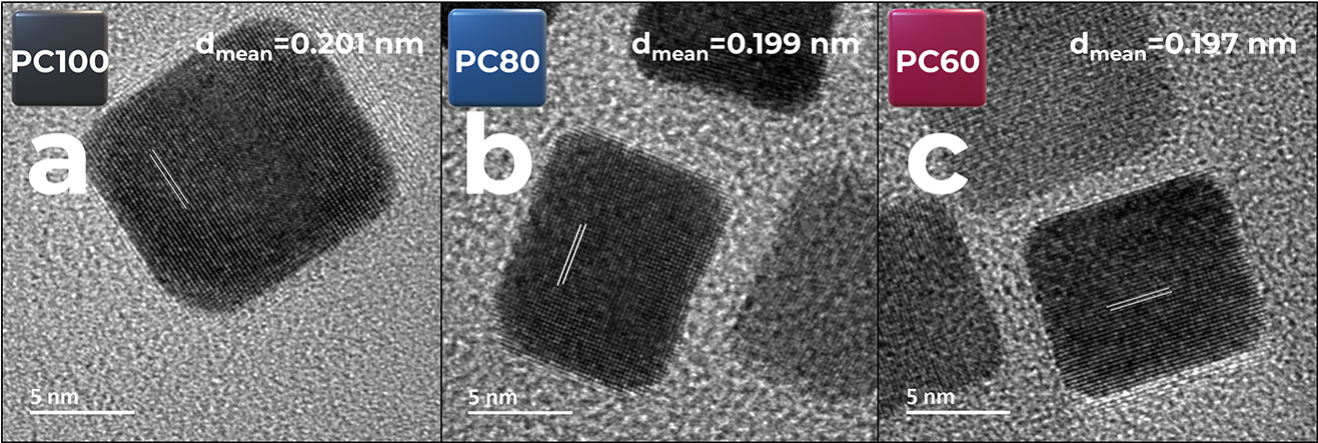
HRTEM images of individual cubic (a) PC100, (b) PC80, and (c) PC60 NPs.

Cubic Pt_*x*_Co_100–*x*_ (*x* = 100, 80, and 60) NPs (PC100, PC80, and PC60), enclosed by {100} faces, respectively, were prepared by using optimized synthesis procedures illustrated in Scheme 1. The (HR)TEM images and size distributions in Figures 1 and S1 show that the synthesized materials have a well-defined cubic shape and a relatively narrow size distribution. The mean particle size was found to be 10.5 ± 1.6, 9.9 ± 2.0, and 9.5 ± 1.5 nm for PC100, PC80, and PC60, respectively (Figure S1d–f). The elemental analysis by TEM-EDS confirmed the desired composition, and the *d*-spacing values slightly decreased with the degree of alloying (Table 1), which were close to *d*_200_ ≈ 0.195 nm of FCC Pt (PDF #04-001-2680). The PXRD illustrated (Figure S2) that all catalysts had diffraction peaks that can be allocated to (111), (200), (220), (311), and (222) planes, with a slight shift indicating Pt alloying with Co. However, only PC80 and PC60 had an intense (200) peak.

**Table 1:** Physical Characterization Data for Pt_*x*_Co_100–*x*_ NPs

catalyst	PC100	PC80	PC60
at % Pt	100	79.3 ± 0.8	56.9 ± 0.7
at % Co	0	20.7 ± 0.5	43.1 ± 0.6
*d*_HRTEM_, nm	0.201	0.199	0.197
*d*_PXRD (200)_, nm	0.1959	0.1956	0.1935

### 3.2 Electrocatalytic Performance toward the Glycerol Oxidation

**Figure 2:**
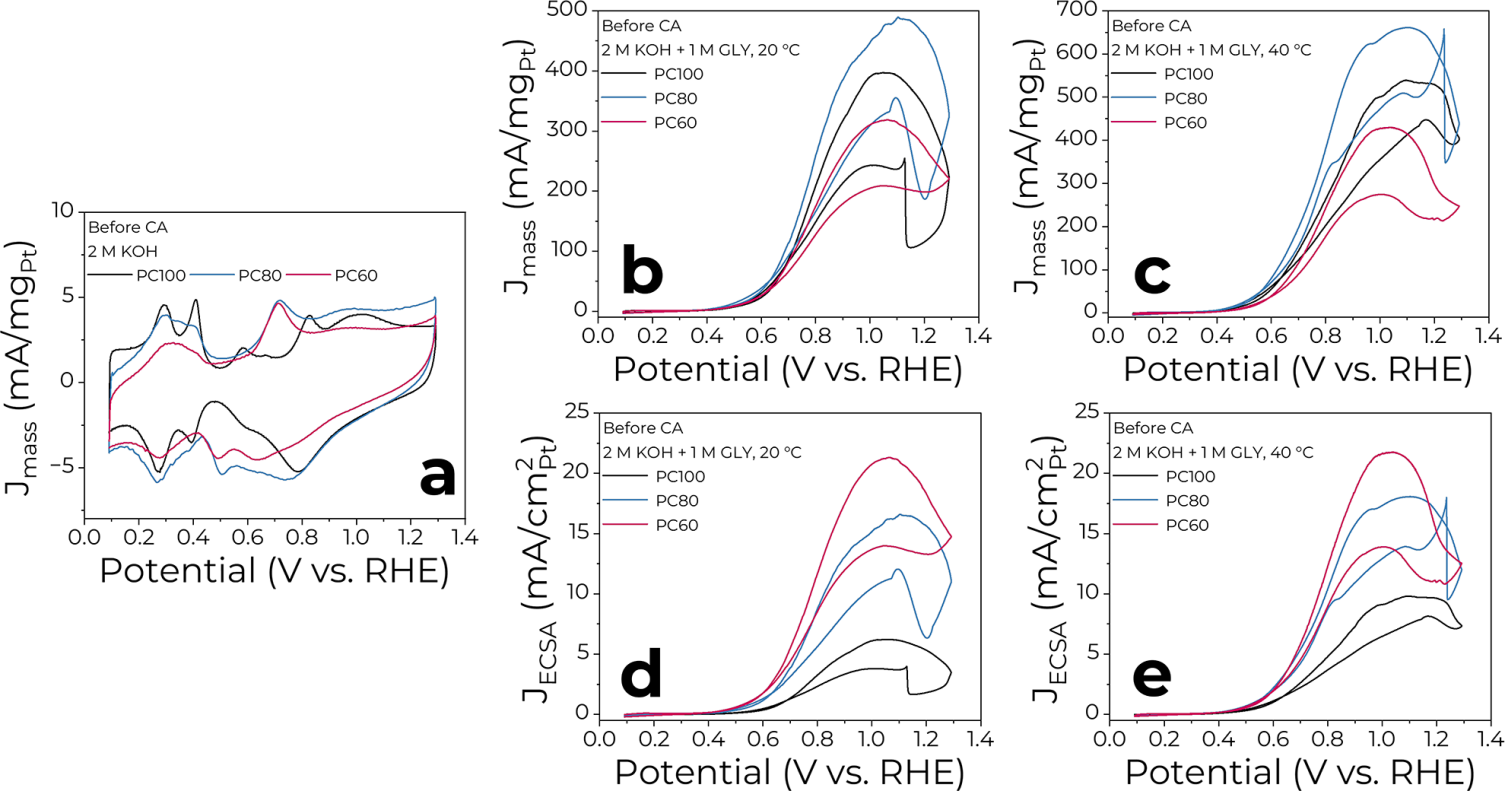
CV profiles of Pt_*x*_Co_100–*x*_ NPs in (a) 2 M KOH and 2 M KOH + 1 M GLY electrolytes at (b, d) 20 °C and (c, e) 40 °C before the potentiostatic measurements. The currents in parts (b, c) and (d, e) are normalized by the absolute Pt mass loading and aECSA, respectively.

The electrocatalytic performance of PC100, PC80, and PC60 and the selectivity of the GEOR were investigated in 2 M KOH and 2 M KOH + 1 M GLY electrolytes at 20 and 40 °C. The results were compared to the Pt_CUBE_ data presented in our previous study.^29^ Typical cyclic voltammograms (CVs) of PC100, PC80, and PC60 NPs in a 2 M KOH electrolyte before glycerol electrolysis are shown in Figure 2a. Generally, the CV curves of PC100 have a typical profile of Pt-based materials with definite peaks in the hydrogen adsorption/desorption (∼0.15 to 0.45 V) region and in platinum oxide film layer formation (∼0.8 to 1.4 V) and reduction (∼1.1 to 0.45 V) regions. For PC80 and PC60, the CV profile exhibits some features similar to PC100; however, the area of the hydrogen adsorption/desorption regions is significantly lower, indicating a decrease in the effective Pt area exposed. In addition, the peaks in the platinum oxide film layer formation and reduction are shifted and possess a new redox pair at 0.7 V/0.5 V that could be assigned to the formation and reduction of a Co(OH)_2_ layer, respectively, or even a redox processes involving metallic Co.^50,51^ The CV profiles in 2 M KOH electrolyte after the glycerol electrolysis (Figure S3a,b) at 20 and 40 °C exhibit an altered shape characteristic of pure Pt, which might occur due to Co cobalt dissolution and formation of a so-called Pt-skin.^51,52^ A strong peak at ∼0.7 V is also observed on PC100, which corresponds to the chemisorption of hydroxide anions on Pt after a period of glycerol electrolysis. The CVs of PC100, PC80, and PC60 in 2 M KOH + 1 M GLY electrolyte before glycerol electrolysis are presented in Figure 2b,c. The typical shape of GEOR with two oxidation peaks in forward and backward scans is observed. The mass activity values reached ∼490 mA/mg_Pt_ at 20 °C and ∼660 mA/mg_Pt_ at 40 °C on PC80 and followed the PC80 > PC100 > PC60 trend. After the glycerol electrolysis, the trend remained the same, and the highest mass activities reached were ∼380 and ∼660 mA/mg_Pt_ on PC80 (Figure S3c,d).

Next, the intrinsic activity of the PC100, PC80, and PC60 catalysts was compared. The currents were normalized by their corresponding aECSA (Figures 2d,e and S3e,f). Here, the previously mentioned electrocatalytic activity trend changed to PC60 > PC80 > PC100. The maximum specific activities before the glycerol electrolysis were observed on PC60 at both temperatures with values of ∼22 mA/cm^2^_Pt_, which was ∼4 times larger than that of PC100. Hence, the incorporation of Co enhanced the performance of Pt-based catalysts. However, after glycerol electrolysis, PC80 and PC60 exhibited a decline in the specific activity, while PC100 remained relatively stable.

As mentioned, the potentiostatic glycerol electrolysis was performed at 0.67 and 0.87 V. The corresponding CA curves in Figure S4 show a typical current decrease that progressively stabilized around 11–20 mA/mg_Pt_ at 0.67 V and 25–60 mA/mg_Pt_ at 0.87 V.

### 3.3 HPLC Results Evaluation

The GEOR selectivity was further evaluated with HPLC analysis of the anolyte samples, as discussed in Sections 2.4–2.5. The analysis confirmed the presence of glycerate, lactate, tartronate, glycolate, glyoxylate, oxalate, acetate, and formate (Figure S5), whereas no traces of dihydroxyacetone, glyceraldehyde, and hydroxypyruvate were detected.

### 3.4 Selectivity toward C3–C1 Products and Glycerol Conversion

A detailed analysis of the GEOR product yield after the glycerol electrolysis on PC100, PC80, and PC60 is presented in Figure S6a,b. It can be seen that the main products—glycerate and lactate—have the highest yield up to ∼45 mM at 20 °C and ∼65 mM at 40 °C, with the maximum yield of ∼58 and ∼63 mM for glycerol and lactate, respectively, observed for PC80.

A comparison of glycerol conversion on PC100, PC80, and PC60 is shown in Figure S6c. The conversion increased with Pt content, applied potential, and reaction temperature, reaching a maximum of 66 mM on PC100 and PC80 at 0.87 V and 40 °C. Nevertheless, the glycerol conversion had repeatedly shown a limit of ∼32 to 35 mM on PC100 at other conditions. The phenomenon might be explained by the possible deactivation of Pt.

**Figure 3:**
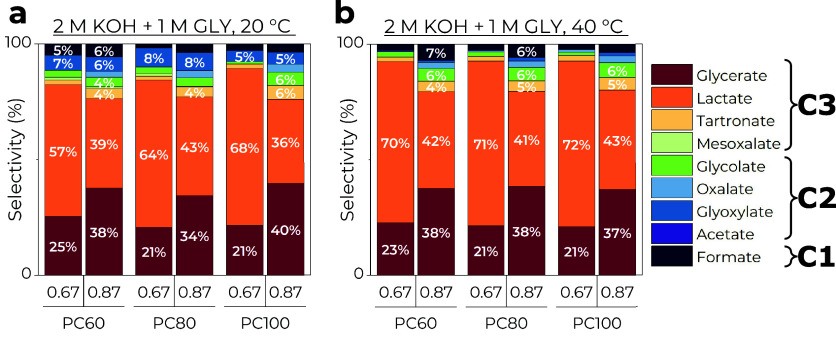
GEOR products selectivity after 2 h of electrolysis at 0.67 and 0.87 V performed at (a) 20 and (b) 40 °C on Pt_*x*_Co_100–*x*_ NPs. Note that product selectivity less than or equal to 3% is not shown.

As can be seen from Figure 3 above, the fractions of GEOR products were not strongly affected by the catalyst composition but were dependent on the potential applied, as expected, with the reaction temperature having an intermediate effect. Despite introducing the non-noble metal Co, the selectivity toward C3 products was preserved. Generally, lactate was a dominant product at 0.67 V with the highest selectivity of 72% on PC100, while formation of glycerate and other C2–C1 oxidation products was facilitated more at 0.87 V as the C–C cleavage was enhanced. Interestingly, the glyoxylate formation is the highest at 20 °C and 0.87 V and almost nonexistent at 40 °C, probably due to the reaction rate not being high enough to oxidize this intermediate quickly.

**Figure 4:**
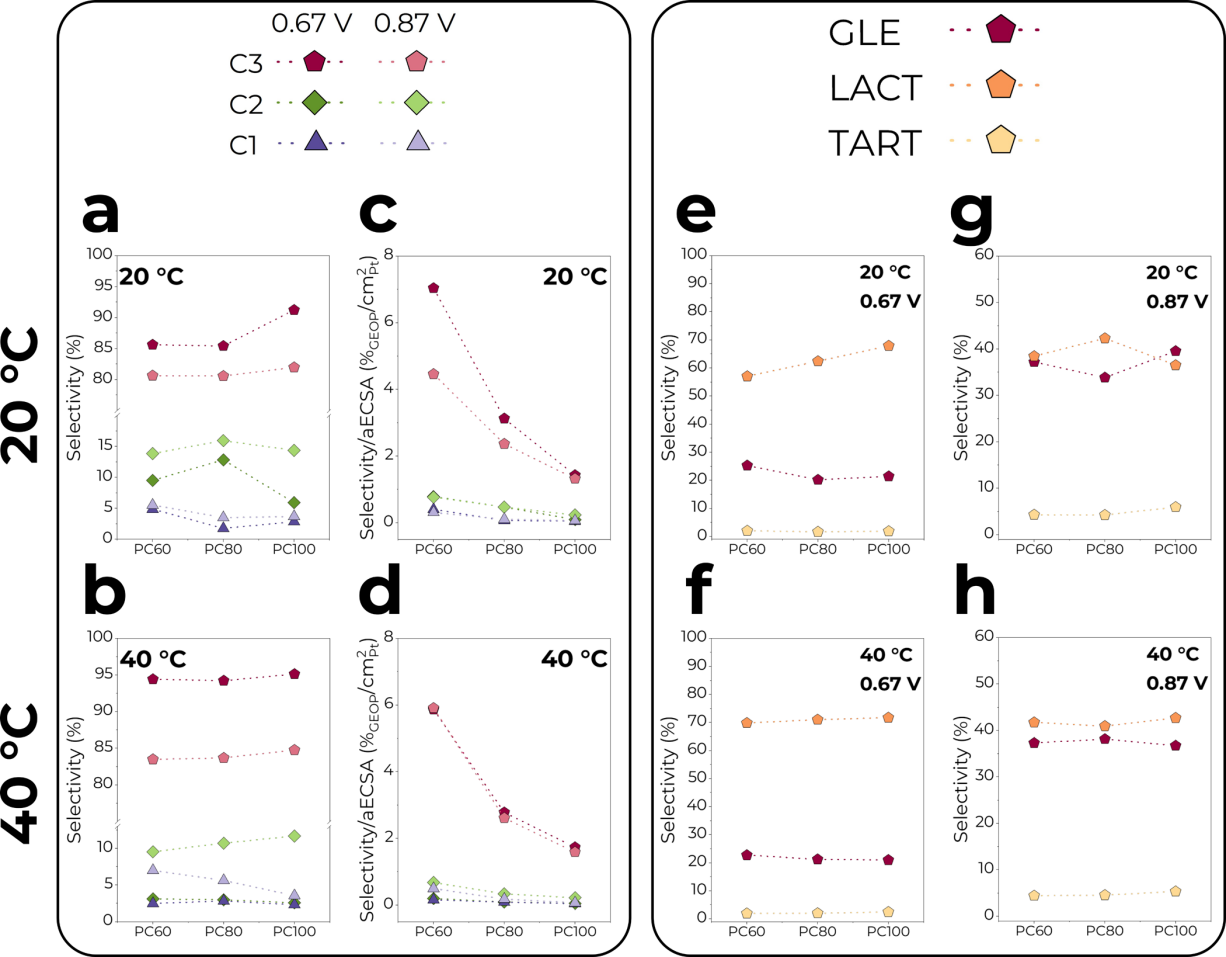
C3–C1 and individual C3 products selectivity as a function of catalyst composition at (a, c, e, g) 20 °C and (b, d, f, h) 40 °C. Note that (c, d) has different *y*-axis scales, where the C3–C1 product selectivity is normalized by the aECSA.

A closer look at the trends is illustrated in Figure 4. The dependency of the total C3–C1 product selectivity on the catalyst composition and reaction temperature is shown in Figure 4a,b. The selectivity of ∼91 and 82% for C3 products was observed on PC100 at both temperatures, being the highest at 0.67 V, where the C–C cleavage is minimal. However, the difference between PC100, PC80, and PC60 was not that dramatic at 40 °C (Figure 4b). The total fraction of C2 products of ∼16% was found to be the highest on PC80 at 20 °C. As mentioned earlier, the effect of Co is fortunately not strong enough to greatly enhance C2–C1 product formation. However, PC60 emerges as the most active catalyst for the C3–C1 products generated per Pt exposed area, as seen in Figure 4c,d. At both temperatures, C3 products are generated on PC60 about 3 times more efficiently than on PC100, making PC60 the most active catalyst intrinsically.

**Figure 5:**
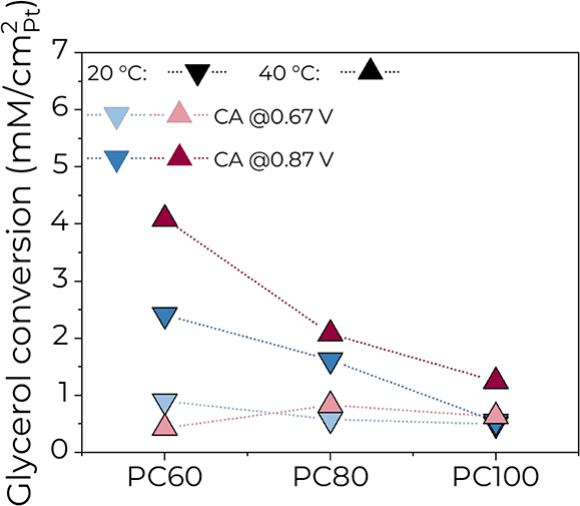
Glycerol conversion as a function of catalyst composition at 0.67 and 0.87 V and 20 and 40 °C in 2 M KOH + 1 M GLY electrolyte. Note that the conversion is normalized by aECSA.

Similarly, when the glycerol conversion is normalized by the aECSA (Figure 5), it changes only slightly between PC100, PC80, and PC60 when the potential of 0.67 V is applied, while the difference is larger at 0.87 V. The conversion per active Pt area was the highest for PC60 at both 20 and 40 °C, again confirming that the combination of Pt with Co improves the electrocatalytic performance.

### 3.5 Selectivity toward Individual C3 Products—Glycerate, Lactate, and Tartronate

The individual C3 product selectivities for PC100, PC80, and PC60 are plotted in Figure 4e–h. As previously seen in Figure 3, lactate was the dominant GEOR product at 0.67 V at both temperatures, and its selectivity increased with increasing Pt content and was inversely proportional to glycerate formation. However, when the potential of 0.87 V was applied, the gap between the selectivities of lactate and glycerate was minimized. In general, glycerate selectivity is highest for PC60. At the same time, tartronate selectivity is relatively stable, indicating that the oxidative dehydrogenation (ODH) of glycerate to tartronate and other C2–C1 products is not strong in highly alkaline media on Pt-based catalysts, and it was seen in our previous study.^29^ The optimal conditions for selective lactate generation via the GEOR, therefore, include a low applied potential and high reaction temperature.

When the intrinsic activity of PC100, PC80, and PC60 for the formation of glycerate, lactate, and tartronate is compared, PC60 was up to 4.7 times better than pure PC100 at both temperatures, which is illustrated in Figure S7a–d.

### 3.6 Insights into the GEOR Mechanism

**Scheme 2:**
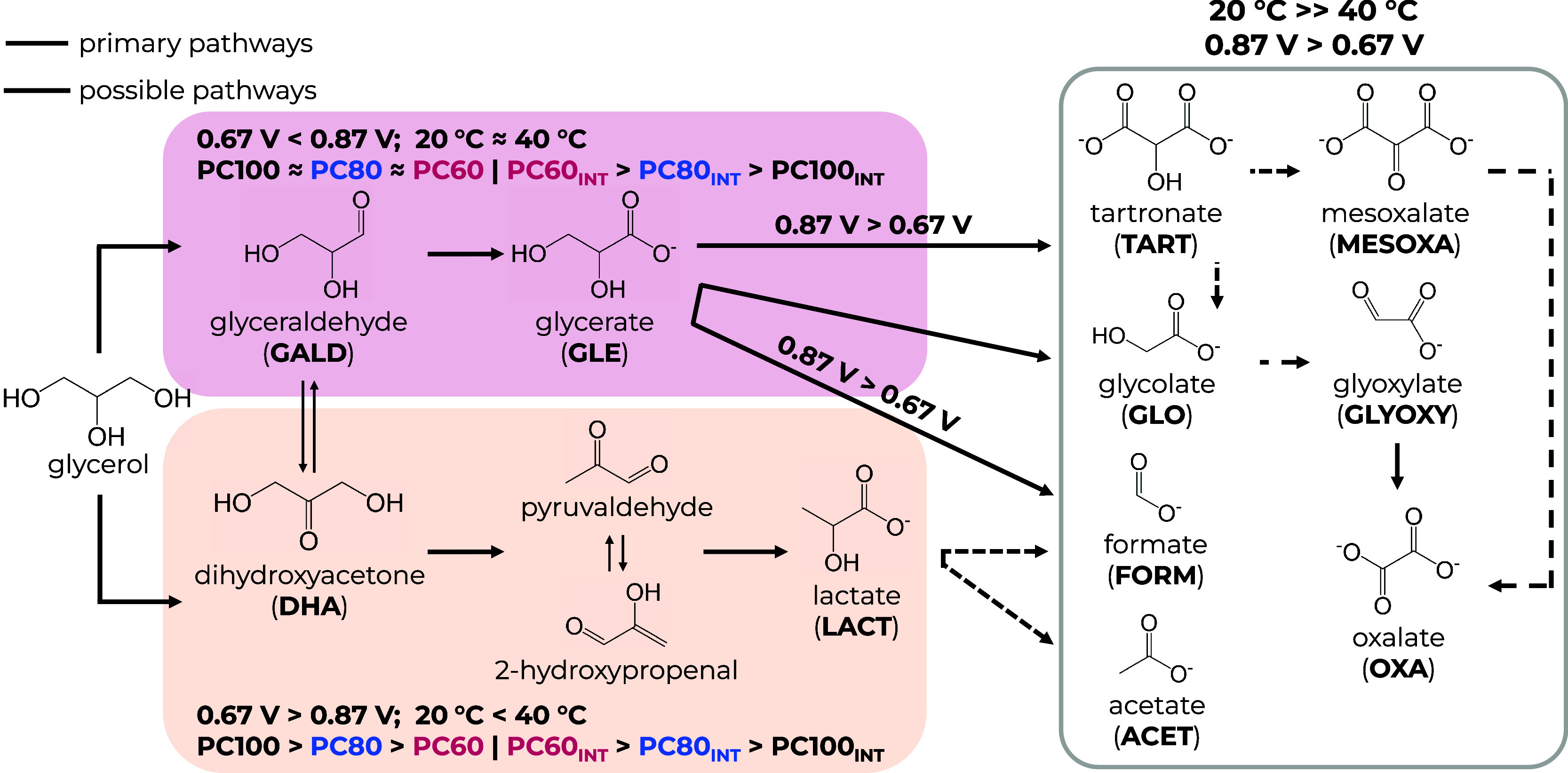
Proposed GEOR Mechanism on Pt_*x*_Co_100–*x*_ NPs in an Alkaline Medium INT = intrinsically.

In general, the mechanism of glycerol (electro)oxidation proceeds as follows: after the initial ODH of glycerol, the reaction can proceed via two general pathways (Scheme 2)—oxidation of a hydroxyl group on either the primary or secondary carbon leads to the formation of GALD or DHA, respectively, which exist in a dynamic equilibrium. As both GALD and DHA are unstable in alkaline conditions, they are rapidly converted to other compounds. From this point, depending on the reaction conditions, the reaction can proceed in two competitive ways: a sequential ODH to GLE or a dehydration to LACT via an intramolecular Cannizzaro rearrangement of pyruvaldehyde (PYRALD). If favored, the C3 intermediates can further undergo an ODH to other C3 products and C–C cleavage to such C2–C1 products as TART, MESOXA, GLO, OXA, GLYOXY, ACET, FORM, and even CO_2_.

In comparison to the present study, multiple reports on GEOR using noble-based nanocatalysts in alkaline media have shown diverse results on GEOR selectivity and dominant GEOR products (Table S3). Zhou et al.^44^ explored the effectiveness of Pd and Pt@Pd nanocubes in the GEOR and examined the impact of applied potential in an alkaline environment at room temperature. Their investigation revealed that the combined action of Pd and Pt enhances the cleavage of the C–C bonds. Pt@Pd nanocubes exhibited superior specific activities compared to Pd nanocubes and commercial Pt/C and Pd/C, leading to a glycolate yield of up to 40% at 0.2 V vs SCE after 2 h of electrolysis. However, when the potential was reduced to −0.1 and −0.4 V versus SCE, the glycolate selectivity decreased to 30%, and glyceraldehyde became the predominant product at 40%. This shift indicates the dominance of the ODH pathway for that catalyst, offering the flexibility to adjust the formation of larger fractions of the C3 products. A direct comparison between the different facets was reported by Terekhina et al.^28^ The GEOR was investigated on octahedral, rhombic dodecahedral, and cubic Pd NPs (Pd_OCTA_, Pd_RD_, and Pd_CUBE_), enclosed by {111}, {110}, and {100} faces, respectively. This investigation was carried out in a 1 M KOH + 0.1 M glycerol solution at 20 and 60 °C, applying a potential of 0.86 V vs RHE. Various value-added C3–C1 products were generated, such as glycerate, lactate, tartronate, glycolate, oxalate, and formate. The Pd NPs exhibited moderate activity in C–C bond breakage at 20 °C, with Pd_RD_ showing the highest glycerate selectivity of 49% after 4 h of glycerol electrolysis. Additionally, glycerate C–C bond cleavage to glycolate was favored on {111} and {100}. At 60 °C, the catalysts became more efficient, promoting glycerate oxidation to tartronate on {111}, {110}, and {100}, while favoring glycerate C–C bond cleavage to glycolate on {110}, resulting in a slightly lower glycerate selectivity, peaking at 42% on Pd_OCTA_. Furthermore, the study revealed the contribution of the dehydration pathway (via DHA), which is particularly prominent on {111}. Consequently, the exposed crystallographic planes had a mild effect on the GEOR selectivity with the following trends observed for glycerate selectivity: Pd_RD_ > Pd_CUBE_ > Pd_OCTA_ at 20 °C and Pd_OCTA_ > Pd_CUBE_ > Pd_RD_ at 60 °C. The selectivity toward C3 products was consistent across different catalysts, with Pd_OCTA_ and Pd_CUBE_ NPs achieving over 50% selectivity at 20 °C and nearly 70% at 60 °C. Glycerol conversion rates were highest for Pd_OCTA_ (21% at 20 °C and 82% at 60 °C), followed by Pd_CUBE_ (10% at 20 °C and 49% at 60 °C), while Pd_RD_ exhibited the least activity, with less than 3% conversion at 20 °C and 35% at 60 °C. Consequently, the optimal conversion rate and C3 product generation ratio followed the sequence Pd_OCTA_ > Pd_CUBE_ > Pd_RD_, and when normalized by the aECSA, the trend was Pd_CUBE_ > Pd_OCTA_ > Pd_RD_. Another study was reported by White et al.^38^ on the effect of the alloying Ni into Pd to form PdNi NPs. PdNi/C exhibited notably enhanced specific and mass activities compared to Pd/C, as assessed by *iR*-corrected polarization curves (ICPCs). Moreover, PdNi/C demonstrated the ability to operate at higher potentials in comparison to Pd/C before deactivation. Density functional theory (DFT) calculations revealed consistently lower potential determining step (PDS) values for PdNi compared to Pd, which aligns with the electrochemical findings. Both PdNi/C and Pd/C exhibited high selectivity toward glycolate and lactate, with product fractions of approximately 60% and 30%, respectively. As potentials increased, the selectivity for glycerate and lactate decreased slightly but remained above 50% and 20%, respectively, with PdNi/C showing slightly higher selectivity for lactate than Pd/C. This study established that bimetallic PdNi NPs are promising electrocatalysts for the GEOR with significantly improved activity compared with monometallic Pd while maintaining selectivity for GEOR products.

In the study by Terekhina and Johnsson,^29^ Pt NPs with cubic (Pt_CUBE_) and dendritic (Pt_DEND_) morphologies were evaluated for the GEOR. The electrocatalytic performance was assessed in electrolytes consisting of 1 M KOH + 0.1 M GLY and 2 M KOH + 1 M GLY at applied potentials of 0.67, 0.77, and 0.87 V vs RHE over 2 h. Glycerol conversion was observed to be in the range of approximately 30–40 mM in both electrolytes. Both Pt_CUBE_ and Pt_DEND_ exhibited high selectivity toward C3 product formation, achieving 86% and 80% in 1 M KOH + 0.1 M GLY and 91% and 87% in 2 M KOH + 1 M GLY electrolytes at 0.67 V, respectively. Notably, in 1 M KOH + 0.1 M GLY electrolyte, C–C bond cleavage was particularly favored at all potentials, especially for Pt_DEND_, owing to their porous structure, which presumably entraps C3 products and facilitates their further oxidation to C2–C1 species. Comparing the selectivity of the dominant C3 products, glycerate and lactate, revealed interesting trends. In the diluted electrolyte, glycerate selectivity decreased in the order 0.67 V > 0.77 V > 0.87 V, while in the concentrated electrolyte, it followed the opposite trend, with a maximum of 43% reached on Pt_CUBE_ at 0.67 V in 1 M KOH + 0.1 M GLY and Pt_DEND_ at 0.87 V in 2 M KOH + 1 M GLY. Regarding lactate, the highest overall selectivity was observed for Pt_CUBE_ and decreased following the sequence 0.67 V > 0.77 V > 0.87 V in both electrolytes, peaking in the 2 M KOH + 1 M GLY electrolyte. The peak selectivity of 68% was achieved at 0.67 V. Pt_CUBE_ exhibited mass and specific activities superior to Pt_DEND_ in both electrolytes. Additionally, Pt_CUBE_ allowed for the highest glycerol conversion and selectivity toward C3 products when normalized to the aECSA. The selectivity of individual C3 products was found to be up to 3 times greater than that of Pt_DEND_. Consequently, Pt_CUBE_ emerged as the top-performing catalyst overall in terms of electrochemical performance and glycerol conversion and selectivity toward the GEOR products. Another investigation into Pt catalysts was conducted by Anil et al.^39^ In that study, Pt catalysts with three distinct pore mesostructures were synthesized and examined, including hierarchical pores (HPC), cubic pores (CPC), and linear pores (LPC). The HPC catalyst exhibited the most promising intrinsic activity due to its optimal combination of large and small pore sizes. Across all three catalysts, glycerate emerged as the primary product, with selectivities reaching up to 60% after 1 h of electrolysis at 0.69 V vs RHE and 60 °C, indicating the promotion of the ODH pathway. A more comprehensive investigation into the GEOR, focusing on its dependency on composition and electrolysis potential, was conducted by Dai et al.^33^ Their study showed that lower applied potentials and higher OH^–^ concentrations enhance lactate selectivity on PtAu/C nanoparticles. The optimum performance was observed for Au-enriched catalysts (Pt_15%surf_Au/C), whereas pure Pt/C nanoparticles exhibited the poorest performance. Conducting GEOR in a solution of 1 M KOH + 0.5 M GLY at 0.45 V vs RHE for 2 h at room temperature resulted in a lactate selectivity of 72%, with the highest glycerol conversion for Pt_15%surf_Au/C. A study by Zhou et al.^32^ investigated a range of binary and ternary alloys comprising Pt-based nanoparticles combined with Ni, Ru, and Rh supported on graphene nanosheets (GNS). The Rh-containing catalysts PtRh/GNS and PtRhNi/GNS exhibited the highest activity toward the GEOR at room temperature. Notably, when the GEOR was conducted at different potentials (−0.4, −0.1, and 0.2 V vs SCE), Pt/GNS displayed the maximum glycolate selectivity of approximately 65% at 0.2 V vs SCE, while PtNi/GNS achieved the maximum glycerate selectivity of about 48% at −0.1 V vs SCE. Furthermore, alloying with Ru was found to facilitate the formation of C3 products, whereas Rh-containing catalysts promoted the formation of C2 products. Overall, the investigated catalysts favored the ODH pathway of the GEOR. Oh et al.^36^ examined the influence of pH on the GEOR using Pt/C and PtCu/C nanoparticles. Generally, PtCu/C showed the highest reaction rate and turnover frequency (TOF) values with an increase in pH. However, the results for pure Pt/C nanoparticles were comparable to those of the present study. For instance, applying a potential of 1.0 V vs RHE at 60 °C, the lactate selectivity increased dramatically from 17 to 37% as the pH rose from 13 to 14, indicating a preference for the dehydration pathway under high alkalinity. Conversely, on PtCu/C, primary alcohol oxidation via the ODH was more prominent than on Pt/C, leading to increased fractions of tartronate and oxalate.

Hence, selecting an appropriate catalyst with suitable properties and optimizing reaction conditions are of great importance for achieving optimal and efficient glycerol electrooxidation.

Based on the discussion in the present study, the following suggestions for the GEOR mechanism on Pt_*x*_Co_100–*x*_ NPs in 2 M KOH + 1 M GLY electrolyte can be made:

1. The GEOR predominantly proceeds via the dehydration to lactate at low applied potentials, whereas the competitive ODH to glycerate is facilitated at higher potentials;
2. Subsequent glycerate ODH to tartronate and C–C cleavage to glycolate, glyoxylate, oxalate, and formate are enhanced at higher applied potentials;
3. The maximum overall selectivity of 94–95% for C3 products is reached at low applied potentials at high temperatures and almost does not depend on the Co content up to 40 at %.

## 4 Conclusions

In the present study, cubic Pt_*x*_Co_100–*x*_ (*x* = 100, 80, and 60) NPs or PC100, PC80, and PC60, respectively, were synthesized, characterized, and, for the first time, investigated as catalysts for the selective GEOR in a highly alkaline medium at both 20 and 40 °C. The mass activity was the highest on PC80 at both temperatures and followed the PC80 > PC100 > PC60 trend, showing an enhanced activity compared to the pure PC100. However, the highest specific activity and intrinsic glycerol conversion were the highest on PC60 and generally followed the PC60 > PC80 > PC100 trend.

The comparison of the GEOR selectivity has shown that despite the high content of Co, even the most Co-rich catalysts, PC60, were highly selective to C3 product formation, reaching up to 94% at 0.67 V. The maximum selectivity of the dominant product, lactate, was found at the applied potential of 0.67 V and was in the range of 57–68% at 20 °C and 70–72% at 40 °C. Meanwhile, the formation of another dominant product, glycerate, was facilitated at the applied potential of 0.87 V and was in the range of 34–40% at 20 °C and 37–38% at 40 °C. At the same time, deeper oxidation of lactate and glycerate to other C3–C1 products was observed at 0.87 V.

Furthermore, the best intrinsic performance was achieved on Co-containing catalysts. The highest selectivity toward C3 products was found for PC60, as well as the selectivity toward individual C3 products (lactate, glycerate, and tartronate), making PC60 the best-performing catalyst overall.

The results support the idea that introducing Co does not hinder the electrocatalytic performance of cubic Pt-based catalysts toward the GEOR. On the contrary, a high Co content improves efficiency even at a high reaction temperature while preserving the selectivity of C3 products. Hence, the present study sets a baseline for developing and optimizing Pt–Co catalysts for the GEOR, which have the potential for practical applications and successful conversion of glycerol into value-added compounds.

## Data Availability

Data will be made available upon reasonable request.

## Supporting Information Available

Low-magnification TEM images, size distribution plots, and PXRD diffractograms of the catalysts; CV profiles of the catalysts after potentiostatic measurements in a 2 M KOH solution, normalized by the Pt mass loading, and in a 2 M KOH with 1 M glycerol solution, normalized by the Pt mass loading and aECSA; chronoamperometric curves, normalized by the Pt mass loading and aECSA; total Faradaic efficiency and carbon balance calculations; HPLC chromatograms of samples aliquoted during the potentiostatic measurements; GEOR products yield and glycerol conversion in mmol/L graphs; and individual C3 products selectivity as a function of a catalyst composition, normalized by aECSA (PDF)
