# Supplementary material for: Improving Glycerol Electrooxidation Performance on Nanocubic PtCo Catalysts
Source: ACS Appl Mater Interfaces. 2024 Oct 14;16(42):56987–96. doi: 10.1021/acsami.4c10219 (PMC11503609; doi:10.1021/acsami.4c10219)
Supplement: Supplementary file 1 — am4c10219_si_001.pdf [file am4c10219_si_001.pdf]

Supporting Information

Improving Glycerol Electrooxidation Performance on Nanocubic PtCo Catalysts

Irina Terekhina[a], Mats Johnsson[a*]

[a] Department of Materials and Environmental Chemistry, Arrhenius Laboratory, Stockholm University, Stockholm SE-106 91, Sweden

[*] Corresponding author

E-mail address: mats.johnsson@mmk.su.se

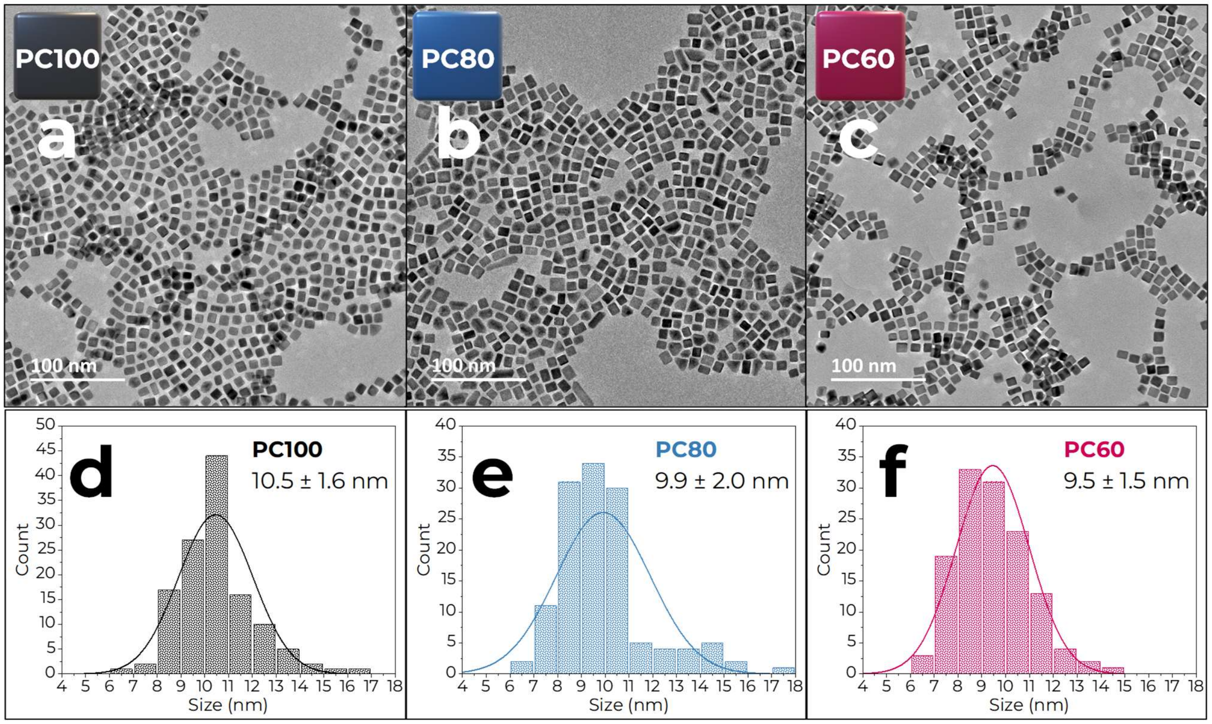

Figure S1. Low-magnification TEM images and size distribution plots for (a, d) PC100, (b, e) PC80, and (c, f) PC60 NPs.

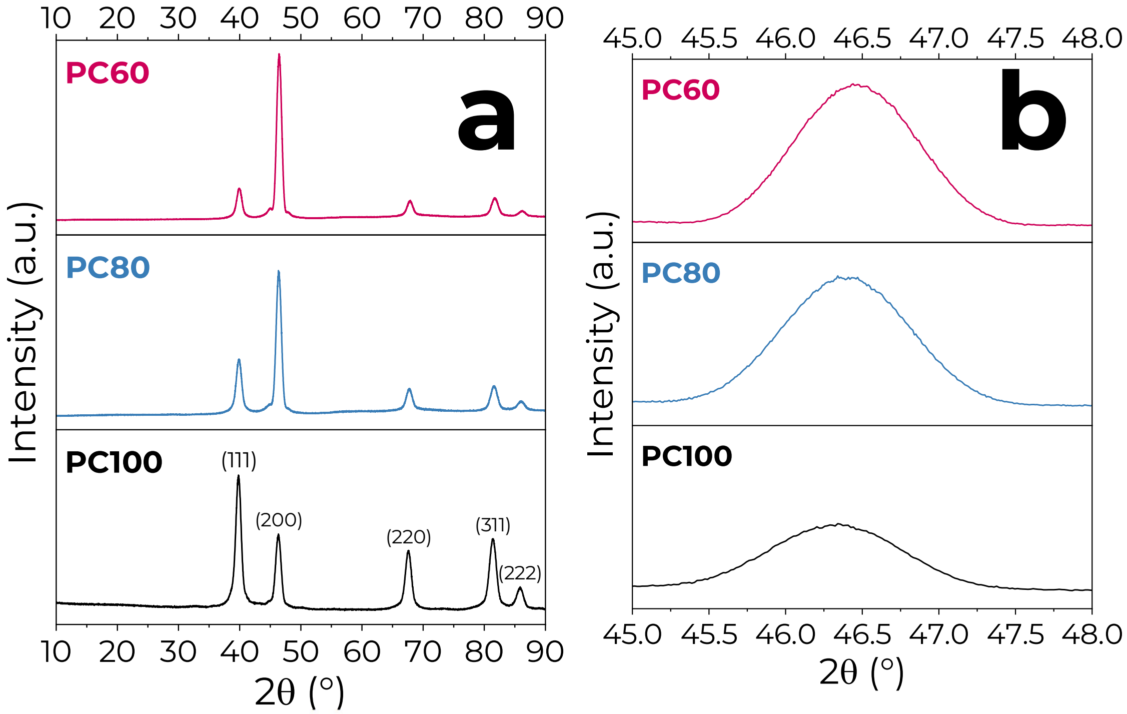

Figure S2. (a) PXRD of Pt_xCo_{100-x} NPs and (b) magnified (200) peaks.

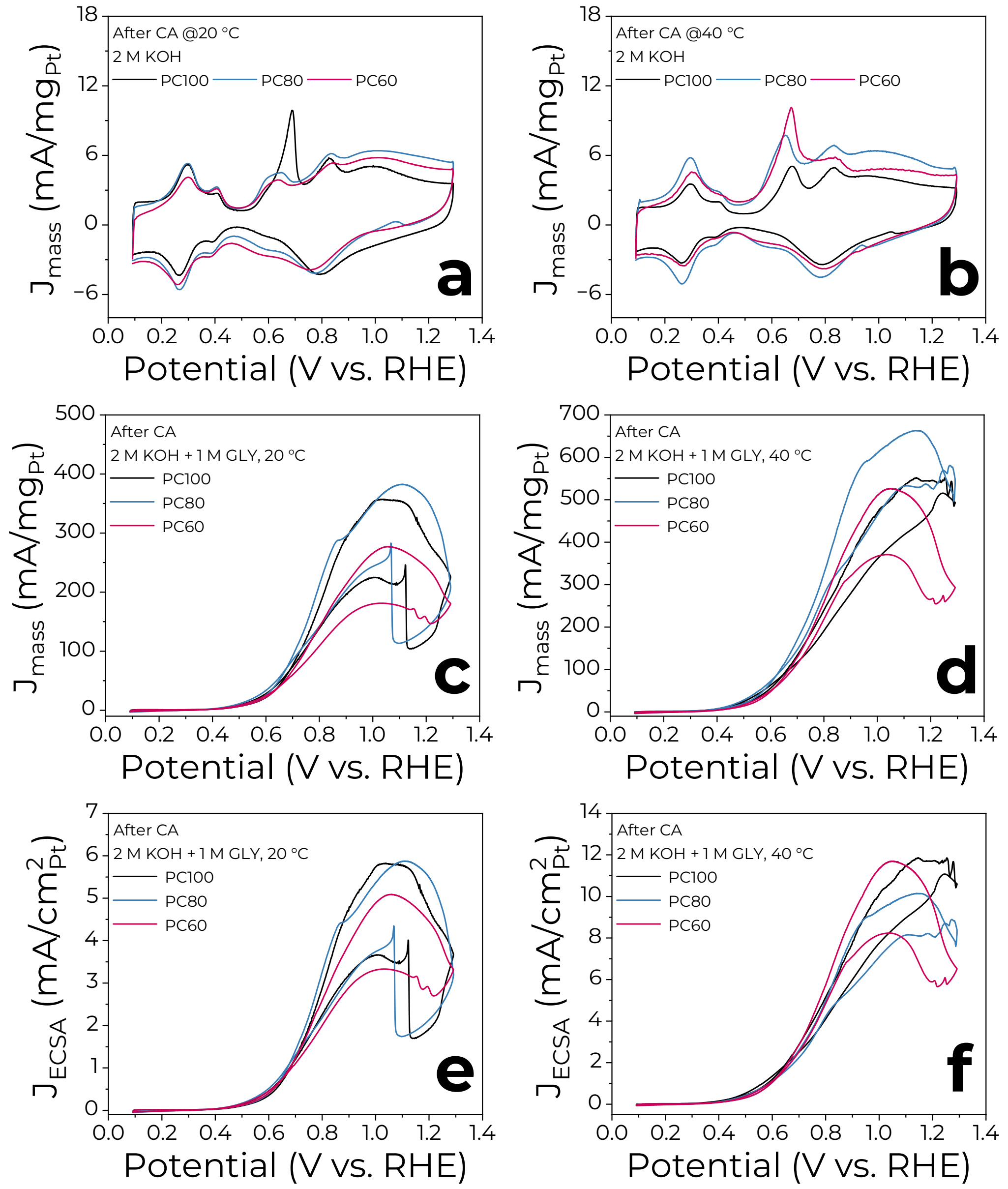

Figure S3. CV profiles of Pt_xCo_{100-x} NPs after the potentiostatic measurements at 20 and 40 °C in (a, b) 2 M KOH and (c–f) 2 M KOH + 1 M GLY electrolytes. The currents in parts (a–d) and (e, f) are normalized by the absolute Pt mass loading and aECSA, respectively.

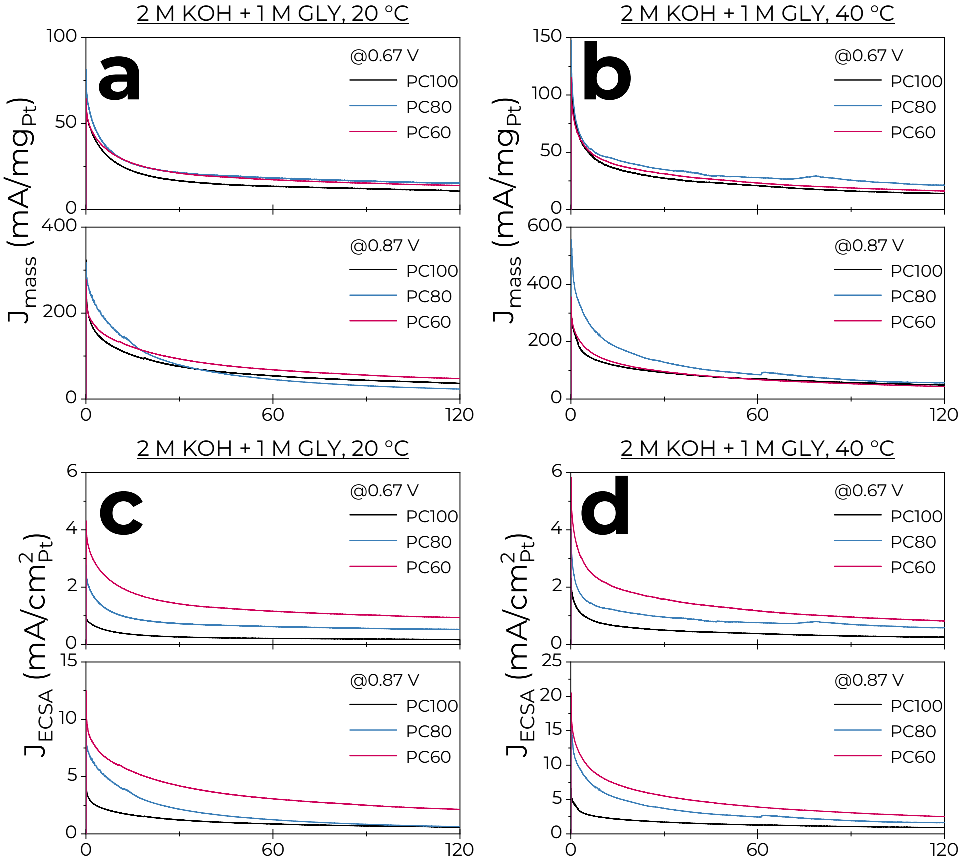

Figure S4. Chronoamperometric curves of Pt_xCo_{100-x} NPs registered in 2 M KOH + 1 M GLY electrolyte at 0.67 and 0.87 V at (a, c) 20 °C and (b, d) 40 °C. The currents in parts (a, b) and (c, d) are normalized by the absolute Pt mass loading and aECSA, respectively.

Product Analysis

The glycerol conversion, product selectivity, carbon balance, and total Faradaic efficiency were calculated using **eqs S1–S4**. The results are presented in **Tables S1–S2**.

Glycerol conversion:

$$\eta_{glycerol} = \frac{C_{0,glycerol} - C_{glycerol}}{C_{0,glycerol}} \cdot 100\% \qquad (\mathbf{S1})$$

where $C_{0,glycerol}$ and $C_{glycerol}$ are the initial and final glycerol concentrations, mmol/l.

Product selectivity:

$$S_{product} = \frac{C_{product}}{\sum C_{all\ products}} \cdot 100\% \qquad (\mathbf{S2})$$

where $C_{product}$ and $C_{all\ products}$ are the individual product and total products' concentrations, mmol/l.

Carbon balance:

$$CB = \frac{C_{glycerol} + C_{C3} + \frac{2}{3} \cdot C_{C2} + \frac{1}{3} \cdot C_{C1}}{C_{0,glycerol}} \cdot 100\% \qquad (\mathbf{S3})$$

where C_{C3} is the concentration of the three-carbon products (glycerate, lactate, tartronate, mesoxalate); C_{C2} is the concentration of the two-carbon products (glycolate, oxalate, glyoxylate, acetate); C_{C1} is the concentration of the one-carbon product (formate). All the concentrations are in mmol/l.

Faradaic efficiency:

Calculations of the total Faradaic efficiency (FE) of the GEOR are based on the following half-reactions:

Glycerate: $CH_2OH\text{-}CHOH\text{-}CH_2OH + 5OH^- \rightarrow CH_2OH\text{-}CHOH\text{-}COO^- + 4H_2O + 4e^-$

Lactate: $CH_2OH\text{-}CHOH\text{-}CH_2OH + 3OH^- \rightarrow CH_3\text{-}CHOH\text{-}COO^- + 3H_2O + 2e^-$

Tartronate: $CH_2OH\text{-}CHOH\text{-}CH_2OH + 10OH^- \rightarrow {}^-OOC\text{-}CHOH\text{-}COO^- + 8H_2O + 8e^-$

Mesoxalate: $CH_2OH\text{-}CHOH\text{-}CH_2OH + 12OH^- \rightarrow {}^-OOC\text{-}CO\text{-}COO^- + 10H_2O + 10e^-$

Glycolate: $CH_2OH\text{-}CHOH\text{-}CH_2OH + 13/2OH^- \rightarrow 3/2CH_2OH\text{-}COO^- + 5H_2O + 5e^-$

Oxalate: $CH_2OH\text{-}CHOH\text{-}CH_2OH + 14OH^- \rightarrow 3/2{}^-OOC\text{-}COO^- + 11H_2O + 11e^-$

Glyoxylate: $CH_2OH\text{-}CHOH\text{-}CH_2OH + 19/2OH^- \rightarrow 3/2HCO\text{-}COO– + 8H_2O + 8e^-$

Acetate: $CH_2OH\text{-}CHOH\text{-}CH_2OH + 7/2OH^- \rightarrow 3/2CH_3\text{-}COO^- + 7/2H_2O + 2e^-$

Formate: $CH_2OH\text{-}CHOH\text{-}CH_2OH + 11OH^- \rightarrow 3HCOO^- + 8H_2O + 8e^-$

$$FE = \frac{\sum z \cdot C_{product}}{Q} \cdot V \cdot F \cdot 100\% \qquad (\mathbf{S4})$$

where z is the number of electrons transferred, V is the anolyte volume, 0.015 L; F is the Faraday constant, 96485 C/mol, and Q is the total charge passed during electrolysis, C.

The carbon balance and Faradaic efficiency were not normalized by the electrolyte evaporation and concentration effects during the electrolysis.

High values above 100% could be due to (i) concentration effects as a result of the electrolyte evaporation, (ii) heterogeneous GEOR on Pt nanocatalysts in addition to the electrochemical oxidation, and (iii) uncertainties in HPLC analysis.

Table S1. GEOR products concentrations, carbon balance, and total Faradaic efficiency of the GEOR for Pt_xCo_{100-x} NPs in a 2 M KOH + 1 M GLY electrolyte at 20 °C at different potentials.

Cat.	Potential (V)	Concentration (mM)									CB	FE
		GLE	LACT	TART	MESOXA	GLO	OXA	GLYOXY	ACET	FORM	%	
PC100	0.67	6.9	21.9	0.6	0.0	0.4	0.0	1.5	0.0	0.9	99.9	109.3
	0.87	38.7	35.7	5.8	0.0	5.7	3.4	5.0	0.0	3.6	105.8	107.7
	**Average**										**102.9**	**108.5**
PC80	0.67	7.8	24.2	0.6	0.5	1.1	0.0	3.1	0.0	0.7	102.1	117.7
	0.87	34.7	43.3	4.3	0.2	3.8	3.1	7.9	0.2	3.6	103.9	116.1
	**Average**										**103.0**	**116.9**
PC60	0.67	8.6	19.4	0.7	0.4	1.0	0.0	2.2	0.0	1.7	102.1	127.3
	0.87	38.3	39.6	4.4	0.7	4.3	2.5	6.4	0.0	5.7	105.0	111.9
	**Average**										**103.6**	**119.6**

Table S2. GEOR products concentrations, carbon balance, and total Faradaic efficiency of the GEOR for Pt_xCo_{100-x} NPs in a 2 M KOH + 1 M GLY electrolyte at 40 °C at different potentials.

Cat.	Potential (V)	Concentration (mM)									CB	FE
		GLE	LACT	TART	MESOXA	GLO	OXA	GLYOXY	ACET	FORM	%	
PC100	0.67	10.6	36.2	1.2	0.0	0.6	0.6	0.0	0.0	1.2	101.5	110.6
	0.87	40.5	47.1	5.9	0.0	7.0	3.3	1.6	0.0	3.9	103.7	92.7
	**Average**										**102.6**	**101.7**
PC80	0.67	12.8	42.9	1.2	0.0	1.1	0.4	0.0	0.0	1.7	103.2	106.9
	0.87	58.5	62.8	7.0	0.1	8.8	4.2	2.2	0.3	8.6	107.5	99.3
	**Average**										**105.3**	**103.1**
PC60	0.67	10.2	31.3	0.9	0.0	1.1	0.1	0.0	0.2	1.1	103.8	107.5
	0.87	37.2	41.6	4.4	0.0	5.5	2.6	0.8	0.0	7.0	103.4	99.6
	**Average**										**103.6**	**103.6**

Table S3. Comparison of recently reported Pt- and Pd-based catalysts for the GEOR.

Ref	Catalyst	Electrolyte	v	E_f	J_{mass} / J_{ECSA}	T	$E_{electrolysis}$	t	$S_{d.prod.}$
**Present work**	PC100	2 KOH + 1 GLY	50	1.05	396 mA/mg_{Pt} 6.2 mA/cm^2_{Pt}	20	0.67 vs. RHE −0.4 vs. SCE	120	lactate (68%) glycerate (21%)
							0.87 vs. RHE −0.2 vs. SCE		glycerate (40%) lactate (36%)
				1.10	539 mA/mg_{Pt} 9.8 mA/cm^2_{Pt}	40	0.67 vs. RHE −0.418 vs. SCE		lactate (72%) glycerate (21%)
							0.87 vs. RHE −0.218 vs. SCE		lactate (43%) glycerate (37%)
	PC80			1.10	486 mA/mg_{Pt} 16.5 mA/cm^2_{Pt}	20	0.67 vs. RHE −0.418 vs. SCE		lactate (64%) glycerate (21%)
							0.87 vs. RHE −0.218 vs. SCE		lactate (43%) glycerate (34%)
				1.10	661 mA/mg_{Pt} 18.1 mA/cm^2_{Pt}	40	0.67 vs. RHE −0.418 vs. SCE		lactate (71%) glycerate (21%)
							0.87 vs. RHE −0.218 vs. SCE		lactate (41%) glycerate (38%)
	PC60			1.06	319 mA/mg_{Pt} 21.3 mA/cm^2_{Pt}	20	0.67 vs. RHE −0.418 vs. SCE		lactate (57%) glycerate (25%)
							0.87 vs. RHE −0.218 vs. SCE		lactate (39%) glycerate (38%)
				1.03	430 mA/mg_{Pt} 21.8 mA/cm^2_{Pt}	40	0.67 vs. RHE −0.418 vs. SCE		lactate (70%) glycerate (23%)
							0.87 vs. RHE −0.218 vs. SCE		lactate (42%) glycerate (38%)
[44]	Pt@Pd nanocubes	0.5 KOH + 0.5 GLY	50	~(−0.13) vs. SCE**	3.2 mA/cm^2_{Pd}	RT*	−0.4 vs. SCE	120	glyceraldehyde (40%) glycolate (30%)
							−0.1 vs. SCE		glyceraldehyde (40%) glycolate (35%)
							0.2 vs. SCE		glyceraldehyde (35%) glycolate (40%)
[28]	Pd nanooctahedra	1 KOH + 0.1 GLY	50	0.93	50 mA/mg_{Pd} 0.9 mA/cm^2_{Pd}	20	0.86 vs. RHE	240	glycerate (29%) formate (24%)
	Pd nanorhombic dodecahedra			0.90	24 mA/mg_{Pd} 1.1 mA/cm^2_{Pd}				glycerate (49%) glycolate (15%)
	Pd nanocubes			0.89	38 mA/mg_{Pd} 1.5 mA/cm^2_{Pd}				glycerate (42%) glycolate (24%)
	Pd nanooctahedra			1.09	183 mA/mg_{Pd} 4.7 mA/cm^2_{Pd}	60			glycerate (42%) lactate (17%)
	Pd nanorhombic dodecahedra			0.87	79 mA/mg_{Pd} 3.9 mA/cm^2_{Pd}				glycerate (27%) formate (26%)
	Pd nanocubes			0.99	155 mA/mg_{Pd} 12.8 mA/cm^2_{Pd}				glycerate (39%) glycolate (16%)
[29]	Pt nanocubes	1 KOH + 0.1 GLY	50	0.92	178 mA/mg_{Pt} 2.9 mA/cm^2_{Pt}	20	0.67 vs. RHE −0.4 vs. SCE	120	glycerate (41%) lactate (40%)
							0.77 vs. RHE −0.3 vs. SCE		glycerate (41%) lactate (30%)

							0.87 vs. RHE<br>−0.2 vs. SCE		glycerate (40%)<br>lactate (22%)
		2 KOH + 1 GLY		1.05	397<br>mA/mg_{Pt}<br>6.2<br>mA/cm^2_{Pt}		0.67 vs. RHE<br>−0.4 vs. SCE		lactate (68%)<br>glycerate (21%)
							0.77 vs. RHE<br>−0.3 vs. SCE		lactate (48%)<br>glycerate (33%)
							0.87 vs. RHE<br>−0.2 vs. SCE		glycerate (40%)<br>lactate (36%)
	Pt dendritic nanoparticles	1 KOH + 0.1 GLY		0.91	93<br>mA/mg_{Pt}<br>0.7<br>mA/cm^2_{Pt}		0.67 vs. RHE<br>−0.4 vs. SCE		glycerate (43%)<br>lactate (29%)
							0.77 vs. RHE<br>−0.3 vs. SCE		glycerate (32%)<br>lactate/oxalate (17%)
							0.87 vs. RHE<br>−0.2 vs. SCE		glycerate (29%)<br>oxalate (22%)
		2 KOH + 1 GLY		1.05	237<br>mA/mg_{Pt}<br>1.9<br>mA/cm^2_{Pt}		0.67 vs. RHE<br>−0.4 vs. SCE		lactate (49%)<br>glycerate (35%)
							0.77 vs. RHE<br>−0.3 vs. SCE		glycerate (39%)<br>lactate (37%)
							0.87 vs. RHE<br>−0.2 vs. SCE		glycerate (43%)<br>lactate (31%)
[39]	Pt catalyst with hierarchical pores	1 NaOH + 0.1 GLY	10	~0.88	~310<br>mA/mg_{Pt}<br>6.9<br>mA/cm^2_{Pt}	60	0.69	60	glycerate (59%)<br>formate (18%)
	Pt catalyst with cubic pores			~0.86	~620<br>mA/mg_{Pt}<br>3.3<br>mA/cm^2_{Pt}				glycerate (58%)<br>formate/oxalate (14%)
	Pt catalyst with linear pores			~0.80	~255<br>mA/mg_{Pt}<br>5.6<br>mA/cm^2_{Pt}				glycerate (60%)<br>formate (17%)
[33]	Pt/C	1 KOH + 0.5 GLY	50	N/A	N/A	RT*	0.45	720	lactate (50%)<br>glycerate (41%)
							0.6		lactate (31%)<br>glycerate (35%)
							0.9		lactate (23%)<br>glycerate (50%)
							1.05		lactate (19%)<br>glycerate (50%)
	$Pt_{90\%surf}Au/C$						0.45		lactate (69%)<br>glycerate (24%)
							0.6		lactate (54%)<br>glycerate (36%)
							0.9		lactate (22%)<br>glycerate (53%)
							1.05		lactate (27%)<br>glycerate (47%)
	$Pt_{64\%surf}Au/C$						0.45		lactate (60%)<br>glycerate (25%)
							0.6		lactate (43%)<br>glycerate (42%)
							0.9		lactate (25%)<br>glycerate (49%)
							1.05		lactate (31%)<br>glycerate (41%)
	$Pt_{29\%surf}Au/C$						0.45		lactate (61%)<br>glycerate (22%)
							0.6		lactate (55%)<br>glycerate (32%)
							0.9		lactate (43%)<br>glycerate (37%)

							1.05		lactate (29%)<br>glycerate (37%)
	$Pt_{15\%surf}Au/C$						0.45		lactate (73%)<br>glycerate (18%)
							0.6		lactate (61%)<br>glycerate (27%)
							0.9		lactate (41%)<br>glycerate (42%)
							1.05		glycolate (33%)<br>lactate (27%)
[32]	Pt/GNS	0.5 KOH + 0.5 GLY	50	−0.03 vs. SCE**	0.3 mA/cm^2_{Pt}	RT*	−0.4 vs. SCE	120	glycolate (36%)<br>glyceraldehyde (32%)
							−0.1 vs. SCE		glycolate (55%)<br>glycerate (15%)
							0.2 vs. SCE		glycolate (65%)<br>glycerate (13%)
	PtNi/GNS			−0.13 vs. SCE**	0.4 mA/cm^2_{Pt}		−0.4 vs. SCE		glycolate (42%)<br>glycerate (36%)
							−0.1 vs. SCE		glycerate (48%)<br>glycolate (33%)
							0.2 vs. SCE		glyceraldehyde (30%)<br>glycerate (28%)
	PtRu/GNS			−0.1 vs. SCE**	0.4 mA/cm^2_{Pt}		−0.4 vs. SCE		glyceraldehyde (31%)<br>glycerate (31%)
							−0.1 vs. SCE		glycerate (40%)<br>glycolate (31%)
							0.2 vs. SCE		glycolate (48%)<br>glycerate (34%)
	PtRh/GNS			−0.16 vs. SCE**	4.5 mA/cm^2_{Pt}		−0.4 vs. SCE		glycolate (41%)<br>oxalate (28%)
							−0.1 vs. SCE		glycolate (40%)<br>glyceraldehyde/ glycerate (19%)
							0.2 vs. SCE		glycolate (52%)<br>glyceraldehyde/ glycerate (14%)
	PtRuNi/GNS			−0.06 vs. SCE**	0.4 mA/cm^2_{Pt}		−0.4 vs. SCE		glycolate (54%)<br>glycerate (26%)
							−0.1 vs. SCE		glyceraldehyde/ glycerate (33%)
							0.2 vs. SCE		glyceraldehyde (39%)<br>glycerate (15%)
	PtRhNi/GNS			−0.15 vs. SCE**	5.6 mA/cm^2_{Pt}		−0.4 vs. SCE		oxalate (38%)<br>glyceraldehyde (31%)
							−0.1 vs. SCE		glyceraldehyde (32%)<br>oxalate (26%)
							0.2 vs. SCE		glycolate (42%)<br>glyceraldehyde (19%)
[36]	Pt/C	0.1 KOH + 0.1 GLY	50	1.02** (RT*)	191 mA/mg_{Pt}<br>0.3 mA/cm^2_{Pt}	60	1.0	N/A	glycerate (59%)<br>lactate (17%)
		0.5 KOH + 0.1 GLY							glycerate (58%)<br>lactate (23%)
		1 KOH + 0.1 GLY							glycerate (49%)<br>lactate (37%)
	PtCu/C	0.1 KOH + 0.1 GLY		1.01** (RT*)	200 mA/mg_{Pt}			N/A	glycerate (62%)<br>lactate (13%)

		0.5 KOH + 0.1 GLY			0.4 mA/cm^2_{Pt}				glycerate (40%)<br>lactate (23%)
		1 KOH + 0.1 GLY							glycerate (45%)<br>lactate (19%)

*room temperature, not specified in °C
**in 0.1 M KOH + 1 M glycerol electrolyte

Electrolyte—electrolyte composition for the potentiostatic measurements, mol/l; v—scan rate, mV/s; E_f—forward peak potential, V vs. RHE if not stated otherwise; J_{mass}—forward peak mass activity, mA/mg_{metal}; J_{ECSA}—forward peak specific activity, mA/cm^2_{metal}; T—temperature, °C; $E_{electrolysis}$—applied potential, V; t—electrolysis time, min; $S_{d.prod.}$—selectivity of the two most dominant products, % (for some references, no exact numerical selectivity values were reported, so they were estimated from the reported GEOR products selectivity plots).

Catalyst supports: C—carbon; GNS—graphene nanosheets. Reference electrodes: RHE—reversible hydrogen electrode; SCE—saturated calomel electrode (Hg/Hg_2Cl_2, sat. KCl).

All quantitative analyses of the GEOR products were performed using High-Pressure Liquid Chromatography (HPLC).

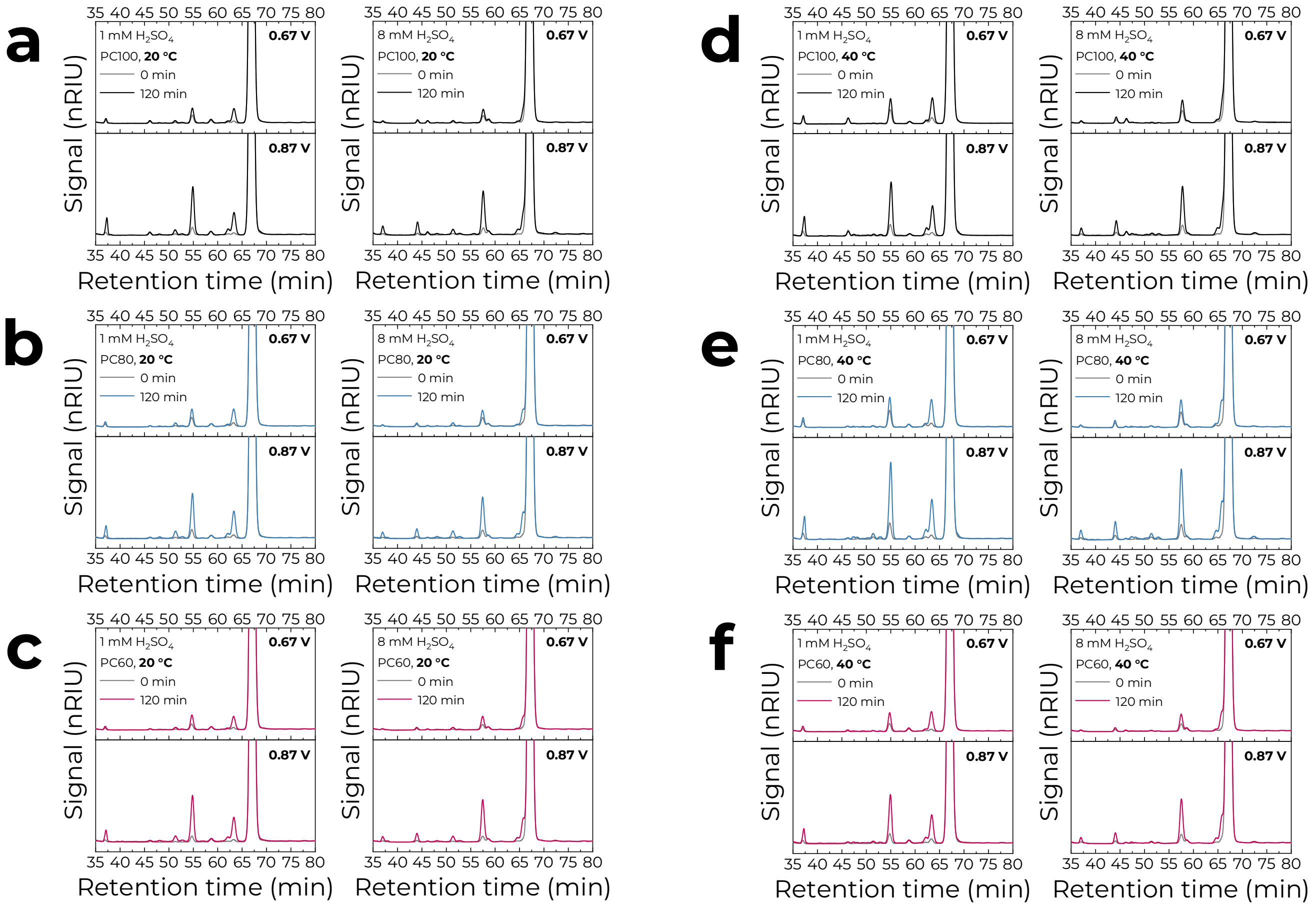

Figure S5. HPLC chromatograms of electrolysis samples collected at (a–c) 20 °C and (d–f) 40 °C and different potentials using 1 mM H_2SO_4 and 8 mM H_2SO_4 as a mobile phase.

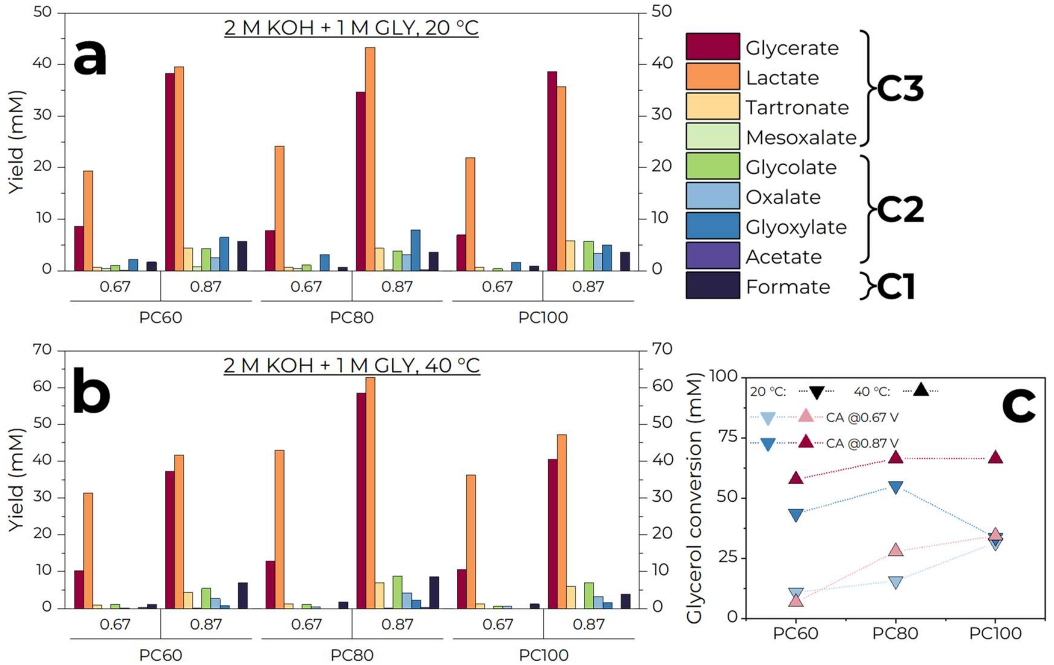

Figure S6. GEOR products yield after 2 hours of electrolysis at 0.67 and 0.87 V performed at (a) 20 °C and (b) 40 °C on Pt_xCo_{100-x} NPs. (c) Glycerol conversion in 2 M KOH + 1 M GLY electrolyte at different applied potentials and temperatures.

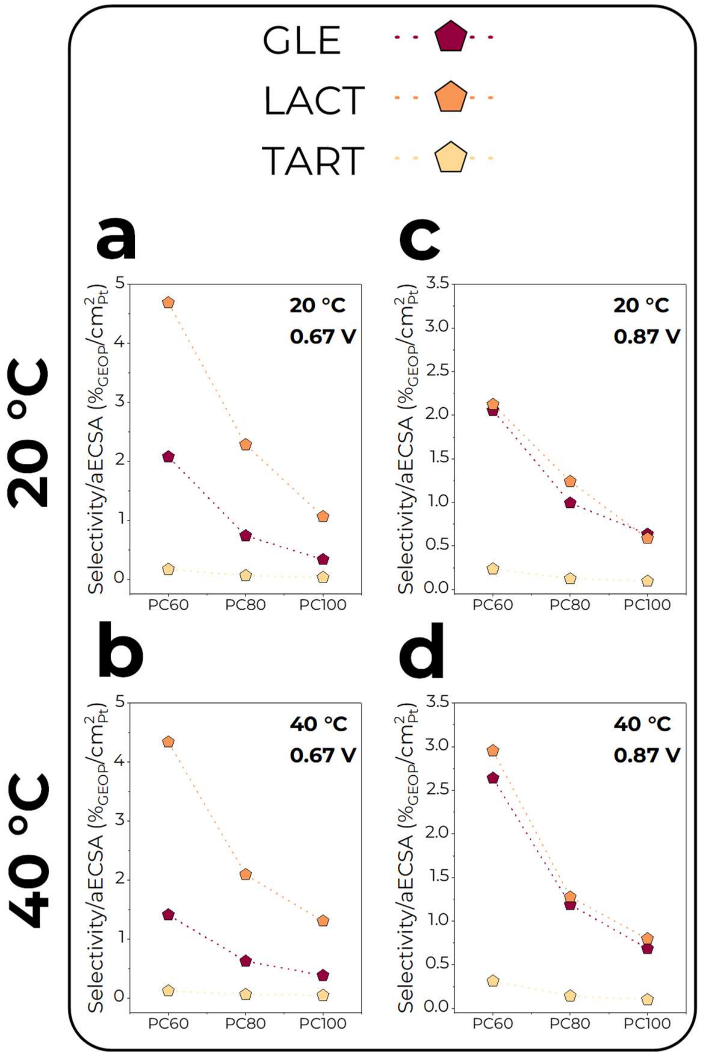

Figure S7. Individual C3 products selectivity as a function of the catalyst composition at (a, c) 20 °C and (b, d) 40 °C normalized by the aECSA, where parts (a, b) and (c, d) correspond to electrolysis at 0.67 and 0.87 V, respectively.
